# pmTR database: population matched (pm) germline allelic variants of T-cell receptor (*TR*) loci

**DOI:** 10.1038/s41435-022-00171-x

**Published:** 2022-04-18

**Authors:** Julian Dekker, Jacques J. M. van Dongen, Marcel J. T. Reinders, Indu Khatri

**Affiliations:** 1 Department of Immunology, Leiden University Medical Center, 2333 ZA Leiden, The Netherlands; 2 Leiden Computational Biology Center, Leiden University Medical Center, 2333 ZC Leiden, The Netherlands; 3 Hogeschool, Leiden, The Netherlands; 4 Delft Bioinformatics Lab, Delft University of Technology, 2628 CD Delft, The Netherlands

**Keywords:** Haplotypes, Immunogenetics, Population genetics, Adaptive immunity

## Abstract

The IMGT database profiles the *TR* germline alleles for all four *TR* loci (*TRA*, *TRB*, *TRG* and *TRD*), however, it does not comprise of the information regarding population specificity and allelic frequencies of these germline alleles. The specificity of allelic variants to different human populations can, however, be a rich source of information when studying the genetic basis of population-specific immune responses in disease and in vaccination. Therefore, we meticulously identified true germline alleles enriched with complete *TR* allele sequences and their frequencies across 26 different human populations, profiled by “1000 Genomes data”. We identified 205 *TRAV*, 249 *TRBV*, 16 *TRGV* and 5 *TRDV* germline alleles supported by at least four haplotypes. The diversity of germline allelic variants in the *TR* loci is the highest in Africans, while the majority of the Non-African alleles are specific to the Asian populations, suggesting a diverse profile of *TR* germline alleles in different human populations. Interestingly, the alleles in the IMGT database are frequent and common across all five super-populations. We believe that this new set of germline *TR* sequences represents a valuable new resource which we have made available through the new population-matched *TR* (pmTR) database, accessible via https://pmtrig.lumc.nl/.

## Introduction

**Fig. 1:**
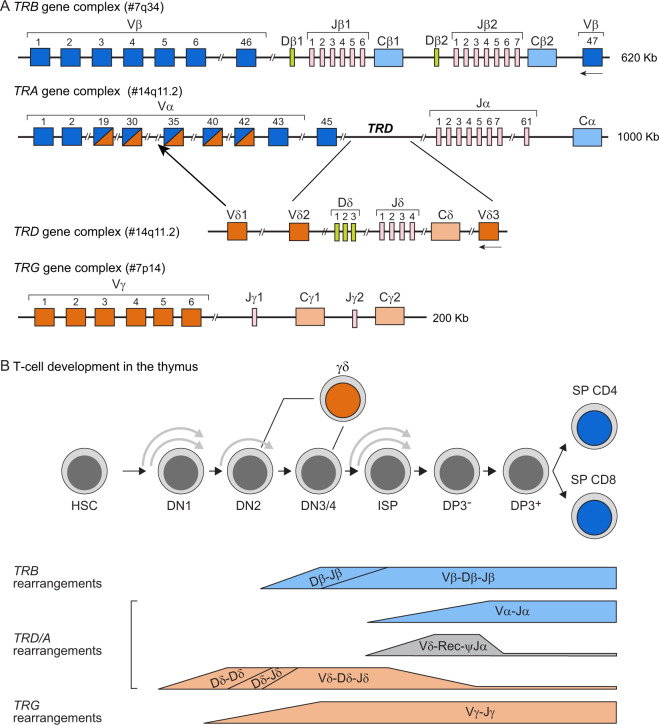
*TR* loci organization and gene rearrangements during human lymphoid differentiation. **A** Schematic overview of the four *TR* loci: *TRB*, *TRA*, *TRD and TRG*. Genomic position of the loci is indicated in brackets. In *TR* loci each rectangle depicts one of the variable (*V*), diversity (*D*), joining (*J*) and constant (*C*) genes. The number of known functional genes, as listed in the IMGT, is indicated underneath each scheme. **B** Overview of *TR* gene rearrangements during human precursor T-cell differentiation in the thymus. HSC hematopoietic stem cell, DN double negative thymocyte, DP double positive thymocyte, ISP immature single positive thymocyte, SP single positive thymocyte. The figure was adapted from Langerak et al. [38].

The genomic organization of four loci of T-cell receptors (*TR*), i.e., alpha (*TRA*), beta (*TRB*), gamma (*TRG*) and delta (*TRD*), is complex. The four loci are distributed over three different genomic regions across two chromosomes in the human genome: *TRA* and *TRD* are located intermingled on chromosome 14q11.2 (with *TRD* embedded within the *TRA* locus), and *TRB* and *TRG* on different arms of chromosome 7 (Fig. 1A). The *TRB* and *TRD* loci are comprised of *Variable* (*V*), *Diversity* (*D*) and *Joining* (*J*) genes, whereas *TRA* and *TRG* loci contain *V* and *J* genes only. The hierarchical rearrangements in the *TR* loci starts with *D*-*D*, *D*-*J* and *V*-*DJ* recombinations in the *TRD* locus, followed by *V*-*J* rearrangements in *TRG* locus. Unless a productive TRγδ receptor is formed, the thymocytes continue to rearrange *D*-*J*, followed by V-DJ rearrangement in *TRB* locus and finally by *V*-*J* recombination in the *TRA* loci (Fig. 1B). Based on the genomic organization and the controlled consecutive rearrangement steps, mature T cells express TRγδ and TRαβ in a mutually exclusive fashion. The recombination of *V(D)J* genes has the potential to generate many millions of different TR molecules, each having a unique antigen binding specificity [1]. The *V(D)J* recombination process is directed by “recombination signal sequences” (RSS), short highly conserved DNA stretches, present at each recombination site of the *TR* genes, i.e., downstream to *V*, upstream to *J*, and at both sites of *D* [2, 3].

*TR* genes harbor inter-individual germline allelic variants, causing different individuals to be able to produce different receptors. As these different allelic variants are shared within confined human populations [4], they contribute also to extreme diversity of receptors at the population level [5]. These population-specific germline variations have been shown to introduce varying disease prevalences in specific population [6–10]. For example, in Asian and Caucasian populations, *TRBV17* plays a pivotal role in Influenza A virus specific T‐cell immunity [11]. Consequently, to understand (population-specific) immune responses, a catalog of population-wide observed *TR* alleles is crucial. Till today, there is, however, only one database that reports all alleles for the *TR* loci: the International ImMunoGeneTics information system (IMGT) [12, 13]. But, this database does not report allelic frequencies or population statistics and, moreover, reported alleles are mostly profiled from Caucasian populations [14, 15].

To enrich the catalog of *TR* germline genes with population information, we relied on the “1000 Genomes (G1K)” dataset (https://www.internationalgenome.org/), derived from cell samples of 2548 individuals across five different ethnicities. We are not the first in doing so. Yu et al. created the Lym1k database for immunoglobulin (*IG)* and *TR* loci, also from the G1K data using their AlleleMiner tool [15]. They, however, did not provide any information on the frequency of the (newly) identified alleles and also the link to population information was not retained. Moreover, not all relevant components of each *TR* locus were stored, i.e., they neglected the *D*, *J*, *Constant (C)* genes and the RSS. Also, they were not able to profile all *TR* genes annotated in GRCh38 genome as they used a previous version of the G1K dataset (i.e., a mapping to GRCh37 being liftover to GRCh38).

Here, we identified the alleles for all components of all four *TR* loci, i.e., the *V*, *D*, *J*, *C* genes as well as the RSS, report reliability scores for the differently detected alleles as well as population information of each allele, and present an online accessible database containing this information which we called the “population-matched germline allelic variants of T-cell receptor loci” database; or in short, the pmTR database. To realize this, we have developed an automated pipeline to profile all the *TR* alleles from the G1K data. The pipeline returns the sequences of alleles, frequency of alleles, as well as the population distribution of each allele among 26 different populations profiled in G1K resource. The resulting alleles are manually curated and made available via the online database (https://pmtrig.lumc.nl/), including population information and allelic frequency to provide access to the community. We have also enabled a BLAST search on the database to directly use our germline alleles in further research.

## Results

### Overview of population matched germline *TR* alleles (pmTR) database

We identified population-specific alleles in all four *TR* loci (*TRA*, *TRB, TRG* and *TRD*) using pmAlleleFinder pipeline (see Methods), where each allele is supported by at least four haplotypes for 2548 individuals belonging to 26 populations representing five continents (Table S1). All the alleles identified from G1K are identified to create the pmTR database. We identified two to three times more new alleles than present in the standard reference IMGT database for all the *V* genes of *TRA* and *TRB* loci (Table 1). These alleles were divided into three allele sets (AS1, AS2, or AS3) based on different support levels (see Methods). AS1 are alleles already present in the IMGT database; AS2 are novel alleles that are frequent in the populations (supported by at least 19 haplotypes, i.e., 10 or more individuals); and AS3 are novel but rare alleles (supported by 4 to 18 haplotypes, i.e., at least 2 to 9 individuals). The pmTR database further contains meta information about the alleles such as the support of haplotypes for each (sub)population (Tables S2–S5). We have also identified the RSS for each gene and the corresponding variants for each allele. The heptamers and nonamers in the RSS turned out not to be conserved for most *TR* genes; this will most likely result in lower recombinant frequencies for genes with less conserved RSS [16]. Similar to *IG* pseudogenes [14], we also identified conserved heptamers for *TR* *V* pseudogenes, suggesting the role of RSS in recombination of *V* pseudogenes in T-cell repertoire [8] (Table S6).

**Table 1:** Number of alleles in functional genes in all four *TR* loci in the pmTR and the IMGT database.

	AS1 (pmTR)	AS2 (pmTR)	AS3 (pmTR)	Total (pmTR)	IMGT
*TRAV*	54	75	76	205	87
*TRAJ*	66	18	17	101	73
*TRAC*	1	2	1	4	1
*TRBV*	68	82	99	249	118
*TRBD*	1	0	0	1	3
*TRBJ*	16	1	0	17	16
*TRBC*	1	2	3	6	4
*TRGV*	13	30	23	66	16
*TRGJ*	5	2	1	8	6
*TRGC*	13	8	7	28	13
*TRDV*	5	5	2	12	5
*TRDD*	3	0	0	3	3
*TRDJ*	4	2	0	6	4
*TRDC*	1	0	2	3	1

The pmTR alleles are divided in AS1, AS2 and AS3 allele set categories. Furthermore, a total number of pmTR alleles are also provided

AS1 (Known), AS2 (Frequent) and AS3 (Rare) are major allele set category of pmTR database.

### *V* gene alleles in the IMGT database are partial and are frequently present in all ethnicities

**Fig. 2:**
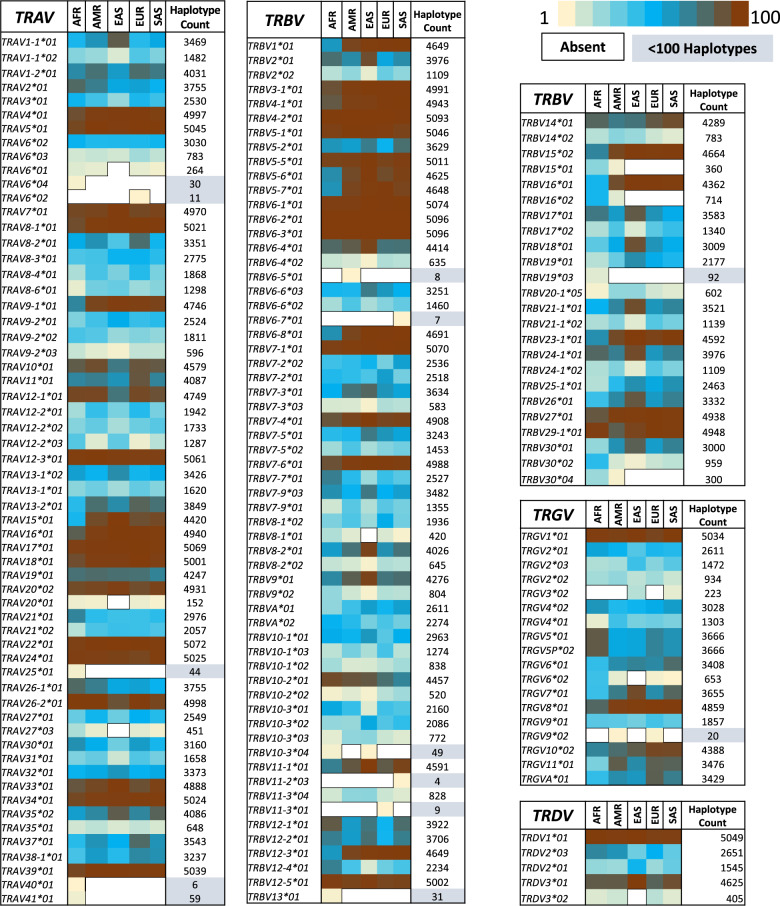
Heatmap representation of the super-population distribution of the known (IMGT) *V* gene alleles in all four human *TR* loci. The IMGT *V* gene alleles identified in the pmTR database are shown here with the IMGT identifiers. The alleles absent in the superpopulations are not colored (white background) whereas the frequency of the alleles in the superpopulation is visualized using heatmap (yellow (0.1%)—Blue (50%)—Brick Red (100%)). The last column shows the haplotype counts of these alleles. The light blue shaded frequencies depict the alleles with <100 haplotypes. Majority of the known alleles are >30% frequent in all the super-populations.

Mapping the pmTR alleles to the known alleles in the IMGT database is instrumental, in this setting, for assessing the frequency and the population specificity of the known alleles, as such information will be helpful in understanding the population-specific response in disease and to vaccines. We found that 60–100% of the IMGT alleles for functional *V* genes in of each *TR* loci were identified in the pmTR database. The functional *V* gene alleles in the IMGT database are not complete as we found 42 of 87 *TRAV* and 53 of 118 *TRBV* alleles to be partial i.e., they do not comprise a leader sequence. In most cases only the first, or first two, alleles of the *TR* genes are sequenced completely. Moreover, the majority of the *TR* genes in the IMGT database had only one allele recorded, implying that a complete *TR* germline allele resource is not available for the research community. When mapping pmTR and IMGT alleles only over the *V* exon region, an increase in the AS1 category alleles was observed (Table 2). We observed that the mutations in the leader region resulted in additional alleles in the pmTR database which could be merged if leader region is not considered for calling these alleles. We believe that leader region also contributes to the selection processes of the immune response, therefore, we will have these alleles called as separate alleles in our resource. Moreover, looking at the super-population distribution of the IMGT alleles in our pmTR database, we found that a majority of these alleles (>90%) are shared among all the super-populations (Fig. 2). Moreover, more than 90% of the mapped IMGT alleles are supported with at least 100 haplotypes and are frequently present in all the super-populations (Fig. 2 and Tables S2–S5).

**Table 2:** Number of pmTR alleles with count of new mutating positions as compared to the existing database i.e. the IMGT database.

Mutating positions	*TRAV*		*TRBV*	
	Complete sequence	*V* region only	Complete sequence	*V* region only
0	66	71	88	104
1	113	114	142	132
2	22	17	14	10
3	3	2	5	3
4	1	1	0	0

Complete sequence includes leader sequence and the *V* region for all the *V* genes. This is important to realize that 42 *TRAV* and 53 *TRBV* alleles in the IMGT database are partial and do not contain the leader sequence.

### The majority of the novel *V* gene alleles are one mutation away from known alleles

To gain information on the number of new polymorphisms added by the novel *V* gene alleles to the variable positions in the known germline alleles, it was important to estimate the differences that are added by the mutations in the Leader region and *V* exon separately as only *V* exon rearranges with *(D)J* genes to generate T-cell repertoire. For complete *V* genes (Leader + *V* exon), we found that 81% (113/139) of the novel *TRAV* alleles and 88% (142/161) of the novel *TRBV* alleles are one mutation away from their known alleles (Table 2), i.e., they only have one different mutation with respect to a known IMGT allele. Based on the alignment of the alleles grouped by gene, we found that these mutated positions are randomly distributed over the *V* gene and do not show specific patterns. We also observed that polymorphisms in ~60% of the novel alleles of *V* genes of all *TR* loci in both AS2 and AS3 category resulted in the non-synonymous changes at the amino acid level. 70% of these genes resulted in one non-synonymous change at the protein sequence level and rest resulted in two non-synonymous changes.

### Novel alleles are unique to super-populations

**Fig. 3:**
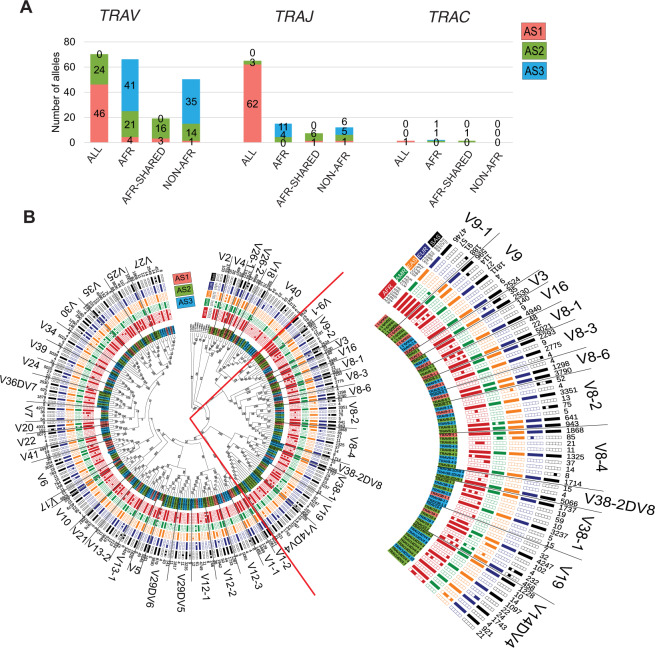
(Super-)population distribution of the alleles in *TRA* loci. **A** The relative super-population distribution of *V, J* and *C* genes for *TRA* locus. The population plots are represented for all super-populations (ALL), Africans only (AFR), Africans shared with one of the other super-populations (AFR-Shared) and “Non-AFR” where alleles are present in one of the populations other than Africans. Red label background indicates AS1 alleles, green AS2 and blue AS3 alleles. The numbers mentioned in the bars correspond to the number of alleles in each category. **B** Maximum Likelihood tree of the population distribution of *TRAV* alleles. Red label background indicates AS1 alleles, green AS2 and blue AS3 alleles. Filled block represents the presence of that allele in at least four haplotypes in that population, otherwise the block is unfilled. A few *TRBV* alleles were used as outgroups. The bootstrap values are mentioned on the branches. The haplotype count of each alleles is mentioned in the outermost circle. The alleles corresponding to each gene is separated by a line and the gene name is mentioned. The zoomed-in view of the tree on the right to the complete tree is visualized.

Opposite to the known alleles (AS1 category), which are shared between all the populations, novel alleles for *TR* loci (AS2 and AS3 categories) are unique to specific super-populations. Remarkably, very few novel *TR* alleles are shared among all five super-populations. One-third of the novel *TRAV* alleles constitute of African-specific alleles (Fig. 3A). Two-third of these novel African-specific alleles are rare, i.e., supported by less than 19 haplotypes. In line with this, 32 of the 34 population-specific *TRAJ* alleles are not known in the IMGT database. Furthermore, we found an uneven diversity in *TRAV* genes, e.g., *TRAV40*, the only known allele of 11 alleles is rare in human populations and we found that each new allele is unique to a particular population (Fig. 3B). Several novel alleles for gene *TRAV8-4*, *V38-1*, *V27* are unique to African populations. Moreover, we also observed frequent novel alleles for *TRAV30* and *TRAV8-2* genes.

**Fig. 4:**
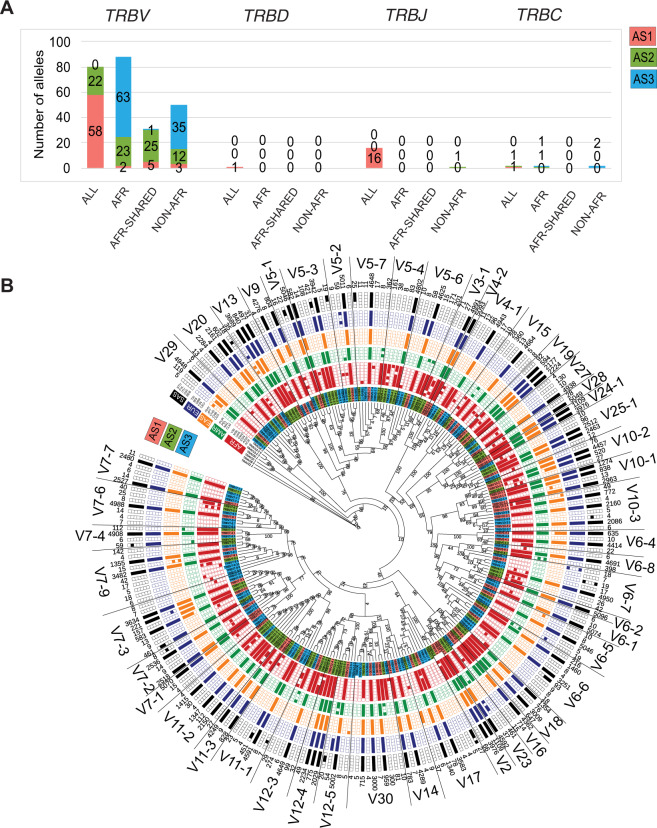
(Super-)population distribution of the alleles in *TRB* loci. **A** The relative super-population distribution of *V, D, J* and *C* genes for *TRB* locus. The population plots are represented for all super-populations (ALL), Africans only (AFR), Africans shared with one of the other super-populations (AFR-Shared) and “Non-AFR” where alleles are present in one of the populations other than Africans for AS1 (red), AS2 (green) and AS3 (blue) alleles. **B** Maximum Likelihood tree of the population distribution of *TRBV* alleles. Red label background indicates AS1 alleles, green AS2 and blue AS3 alleles. The population distribution is plotted in a binary format where each block is a population. Filled block represents the presence of that allele in at least four haplotypes in that population, otherwise the block is unfilled. A few *TRAV* alleles were used as outgroups. The bootstrap values are mentioned on the branches. The haplotype count of each alleles is mentioned in the outermost circle. The alleles corresponding to each gene is separated by a line and the gene name is mentioned.

*TRBV* follows a similar pattern as the *TRAV* alleles; half of the novel alleles are African-specific of which a majority is rarely present in Africans (Fig. 4A). However, we do not observe a similar pattern for the *TRBD*, *TRBJ* and *TRBC* alleles. Similar to *TRAV* population distribution, we observed a specific pattern in diversity of *TRBV* genes. The novel alleles in *TRBV5-4* and *V7-9* genes consists several alleles unique to African populations whereas *TRBV5-6*, *V15* and *V23* genes comprises alleles shared between African and American populations (Fig. 4B). On the contrary, *TRBV6-7* gene comprises novel South Asian alleles. In general, half of the “AFR-Shared” *TRAV* and *TRBV* alleles are common between African and American super-populations (Figs. 3B and 4B), suggesting a role of intermixing due to migratory history of Africans to America [17, 18].

**Fig. 5:**
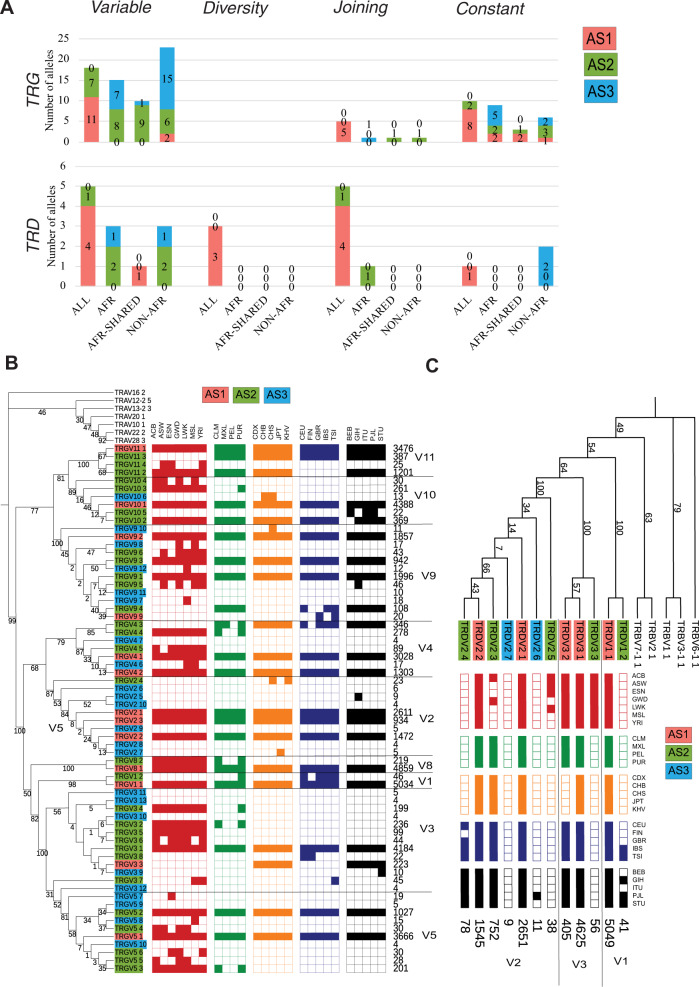
(Super-)population distribution of the alleles in *TRG* and *TRD* loci. **A** The relative super-population distribution of *V, D, J* and *C* genes for *TRG* and *TRD* locus. The population plots are represented for all super-populations (ALL), Africans only (AFR), Africans shared with one of the other super-populations (AFR-Shared) and “Non-AFR” where alleles are present in one of the populations other than Africans for AS1 (red), AS2 (green) and AS3 (blue) alleles. **B** Maximum Likelihood tree of the population distribution of *TRGV* alleles. The population distribution is plotted in a binary format where each block is a population. Filled block represents the presence of that allele in at least four haplotypes in that population, otherwise the block is unfilled. A few *TRAV* alleles were used as outgroups. The bootstrap values are mentioned on the branches. **C** Maximum Likelihood tree of the population distribution of *TRDV* alleles. The population distribution is plotted in a binary format where each block is a population. Filled block represents the presence of that allele in at least four haplotypes in that population, otherwise the block is unfilled. A few *TRBV* alleles were used as outgroups. The bootstrap values are mentioned on the branches. The haplotype count of each alleles is mentioned in the end of the branch. The alleles corresponding to each gene is separated by a line and the gene name is mentioned. Please note that the nomenclature of *TRDV4-8* genes correspond to *TRAV* genes as: *TRDV4* is *TRAV14*, *TRDV5* is *TRAV29*, *TRDV6* is *TRAV23*, *TRDV7* is *TRAV36* and *TRDV8* is *TRAV38-2*.

*TRG* and *TRD*, being the smallest *TR* loci, have fewer novel alleles as compared to the *TRA* and *TRB* loci (Fig. 5A). However, unlike *TRAV* and *TRBV* novel alleles, novel *TRGV* alleles often belong to Non-African populations (Fig. 5A). We found a higher diversity in *TRGV3*, *V5* and *V9* genes as compared to other genes suggesting the role of evolutionary pressure on these genes (Fig. 5B). Moreover, the *TRG* locus has the highest number of alleles for *C* genes across all four *TR* loci, a majority of which belongs to the rare category (AS3) (Fig. 5A). The *TRD* locus is the most conserved locus as very few novel alleles were found for the *TRDV*, *TRDJ* and *TRDC* genes (Fig. 5A). An equal distribution of African-specific and non-African-specific alleles was observed in *TRDV* alleles, wherein the majority of non-African alleles were specific to European and South Asian populations (Fig. 5C). Interestingly, similar to *TRGV*, we also found a more diverse pattern in *TRDV2* gene as compared to the *TRDV1* and *TRDV3*.

Summarizing, we find a similar super-population distribution across all four *TR* loci despite their size difference, peculiar gene-specific population distribution and size of gene subfamilies.

### A majority of non-African alleles are specific to Asian super-populations

**Fig. 6:**
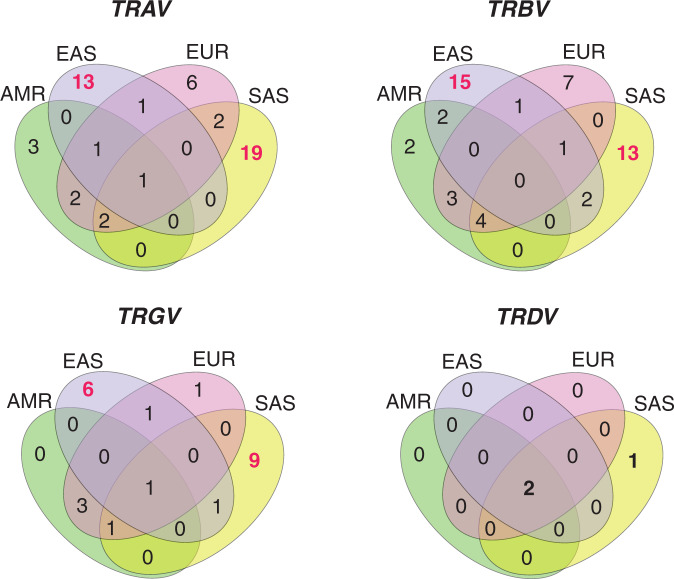
Super-population specificity of the non-African alleles in *TRAV*, *TRBV*, *TRGV*, and *TRDV* alleles. The number of genes specific to East and South Asian populations are shown in red color, showing that Asian populations are more diverse.

The super-population distribution of novel *TRA*, *TRB* and *TRG* alleles show that these are specific to the Non-African populations (Figs. 3–5). In fact, a majority of them belongs to the East and South Asian super-populations (Fig. 6). Interestingly, these alleles are not specific to any of the Asian populations. Very few alleles were shared between Asian, American and European super-populations, suggesting an exclusive nature of the *TR* loci in these ethnicities. The larger number of Asian-specific alleles suggests an exceptional diversity in Asia which went unnoticed in the current databases.

### Validation of novel pmTR alleles using publicly available novel alleles

We observed substantial overlap of our novel alleles (i.e., AS2 category alleles) with the recently published articles profiling the novel *TR* alleles [19–21] (Tables S2–S5). All these studies profiled novel alleles for *TRBV* genes, whereas only Lin et al. reported novel alleles for *V* and *J* genes for all the four *TR* loci. 14 of 123 AS2 and 8 of 108 AS3 *TRAV* alleles from the pmTR database could be mapped to the novel alleles reported by Lin et al. [21] (Table S2). Similarly, 11 of 18 AS2 and 2 of 18 AS3 *TRJV* alleles could be mapped as well. *TRAV* alleles from the pmTR database did not have a huge overlap with the novel alleles from other studies, however, we observed that almost half (50 of 110) of the novel AS2 *TRBV* alleles could be mapped to one of these three studies (Table S3). A few of these mapped *TRBV* alleles (16 alleles) were supported by at least two different resources. Moreover, the African-specific *TRBV* alleles mapped from Luo et al. [19] were also found to be African-specific alleles in our resource. Similarly, 19 of 45 AS2 *TRGV* alleles could be identified in the novel alleles by Lin et al. (Table S4).

### Validation of pmTR alleles using NYGC long read high coverage data

NYGC long read data sequenced the G1K samples with high coverage (30X) and longer read technology (NovaSeq 6000) which has resulted in larger number of SNPs, INDELs and structural variants as compared to the phase 3 G1K dataset [22]. Accordingly, we also observed larger number of polymorphisms henceforth alleles in all the *TR* loci (Table S7). Especially in the category of alleles for functional *TRBV* genes, >100 additional alleles were identified in the NYGC datasets. Similarly, >20 additional alleles were identified for *TRBC* genes which was significantly higher as compared to six *TRBC* alleles observed in phase 3 dataset. Except *TRB* loci, we do not observe such a considerable increase in the number of alleles in the NYGC dataset. Only, 10–30 new alleles for *TRAV* and *TRGV* functional genes were observed. For *TRD* loci, exact number of alleles were observed in NYGC-2504 dataset as observed in phase 3 dataset.

Except the alleles for *TRBC*, *TRGC*, and *TRGV* functional genes, ~80% of the *TR* alleles identified from phase 3 dataset could be identified in NYGC dataset. Only ~50% of *TRBC* and *TRGC* alleles and ~70% alleles for functional *TRGV* genes from phase 3 dataset could match with the alleles identified in the NYGC datasets. A 100% match was observed for *TRAV* pseudogenes, *TRAC*, *TRBD*, *TRBJ*, *TRGJ*, *TRDV*, *TRDD*, *TRDJ* and *TRDC* alleles between phase 3 and NYGC datasets. Additionally, we observed that 18 already known alleles in the publicly available datasets [19–21] were not identified by the NYGC datasets (Tables S2–S5). Majority of these alleles belong to the genes where either none or a single allele matched between phase 3 and NYGC datasets, for example, *TRAV1-2*, *TRAV19*, *TRBV11-2*, *TRBV5-4*, *TRBV5-7*, *TRBV6-5*, *TRBV7-2*, *TRBV7-7*, *TRBV10-2*, *TRGV3*, *TRGV5*, *TRGVB* genes.

While investigating the population distribution of the alleles from NYGC dataset, we observed that although a majority of the phase 3 *TR* alleles were present in the NYGC dataset, the population distribution of the alleles was not retained as found in phase 3 dataset (Fig. S4). Almost 50% or more alleles belonged to the category of all populations and only 8–10% alleles of the total pmTR alleles could be assigned as AFR specific. This was completely in contrast with the population distribution observed in phase 3 dataset where a majority of the alleles were identified to be African-specific with good haplotype support. To exemplify, *TRBV4-1_2* pmTR allele from phase 3 dataset is supported by 96 haplotypes wherein 92 haplotypes are originating from African populations. Moreover the allele is also recorded as African-specific allele by Luo et al. [19]. However, the support for this allele in NYGC dataset is only 10 haplotypes that as well distributed across different populations.

In summary, we observed larger number of alleles in NYGC dataset as compared to phase 3 dataset with a significant overlap with the alleles from phase 3 dataset. However, the population distribution of the alleles has changed drastically suggesting the sample level haplotype phasing is different between phase 3 and NYGC dataset.

### Evolutionary dynamics of all four *TR* loci in different populations

**Fig. 7:**
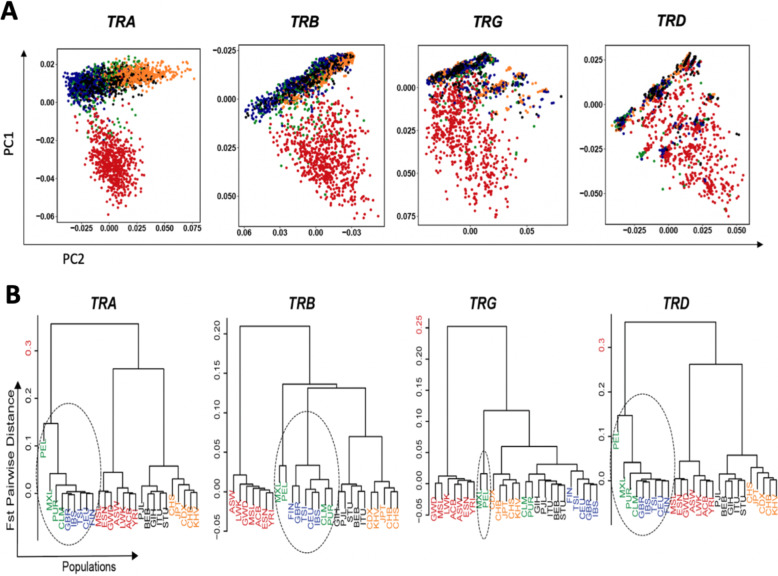
Genetic diversity and population structure in five super-populations for *TR* loci. **A** Separate PCA plot of each of the four *TR* loci based on the polymorphisms in the complete locus. Each dot represents a sample where each sample is colored based on the super-population they belong. **B** Pairwise population distribution calculated by Fst Matrix is represented as a cladogram for each locus namely *TRA*, *TRB, TRG* and *TRD*. Five super-populations are colored as Africans in red; Americans in green; East Asians in orange; Europeans in blue and South Asians in black.

A principle component analysis (PCA) was performed on the entire span of all four *TR* loci to visualize the genetic diversity among different loci. We found that the Africans are highly diverse in all the four loci, whereas other super-populations are comparatively similar to each other (Fig. 7). Interestingly *TRA* and *TRB* follow similar patterns despite being on different chromosomes. On the other hand, in the *TRG* and *TRD* loci, we observed multiple clusters of individuals from all the super-populations having unique variability as compared to the variation in the individuals belonging to African populations. Despite that the *TRD* locus lies within the *TRA* locus, it does not follow a similar variability as the *TRA* locus, implying that selection pressure has been different when comparing the *TRA* and *TRB* loci with the *TRG* and *TRD* loci. This may have been governed by the size of loci and particularly by different functional aspects of the TRαβ vs. TRγδ molecules.

To investigate the population structure in more detail, we calculated the population differentiation for each *TR* loci separately. We found a comparatively different structure between the loci as compared to the genetic diversity assessed using PCA in Fig. 7A. We found Africans to be the most diverse in the *TRB* and *TRG* loci whereas Americans and Europeans are ancestor clades for the *TRA* and *TRD* loci. The population structure is in accordance with the chromosomal organization of these loci (Fig. 7B), unlike the genetic diversity visualized in the PCA plot. Interestingly, in all the *TR* loci, an early separation of Mexicans (MXL) and Peruvians (PEL) populations is observed (Fig. 7B). Similar to the population structure in *TRG* loci, the relatedness of the PEL population was also observed in the *IG* loci [14].

## Discussion

We performed a comprehensive analysis of *TR* germline alleles identified from 2548 individuals available in the G1K data resource belonging to 26 populations across five different ethnicities. The *TR* germline alleles from the G1K resource were also identified previously by Yu et al. and compiled in the Lym1K resource. This resource, however, does not provide information on the allelic frequencies and population-specificities of the alleles [15]. In addition, we not only used a minimum haplotype count (i.e., 4 haplotypes) to identify alleles using our automated pipeline “pmAlleleFinder”, but also categorized the pmTR alleles in three category of alleles. Moreover, potential false-positive germline alleles in operationally indistinguishable (OI) genes were also eliminated by manually assessing the mutating positions between the alleles belonging to OI genes [14]. Finally, the filtered alleles for *TR* loci were compiled in the pmTR database (hosted via https://pmtrig.lumc.nl/). Application of these rules and checks, has provided us with *TR* germline alleles, however, the inherent limitations of the short-read mapping to the reference genome may persist. The mis-mapping of short reads are majorly limited to the duplicated genes (e.g., the genes highlighted in blue in Fig. S2) which can lead to false positives in the allele calls. On the other hand, using a threshold-based cut-off can also lead to true negatives as we might miss individual-specific alleles. Moreover, the low coverage of the reads may potentially impact the allele calls but we know from the previous study that mapping outcomes will not differ at 30, 40 or 50X read coverage [23].

The assignment of the alleles to three categories provided us with support for the pmTR alleles e.g., 60–100% of the IMGT alleles for *V* genes were also recorded in the pmTR database. A complete overlap between the pmTR and IMGT *V* gene alleles is hampered by the number of individuals with which the pmTR database is created, i.e., 2548, as compared to hundreds of individuals profiled by the IMGT database over the last decade. However, in the pmTR database, all genes/alleles from one individual are profiled, whereas the IMGT database has been compiled with the few genes/alleles of many different individuals. We found that >90% of the IMGT alleles are shared between all five super-populations, with very few alleles being specific to one or more of the super-populations, suggesting that the IMGT database lacks rare population-specific alleles. Having only alleles that are shared uniformly and frequently across all super-populations, poses limitations for immune-response studies that aim at understanding the genetic basis of difference between and among different human populations [4, 24].

Furthermore, we observed that a majority of the AS2 alleles of pmTR database especially *TRBV* and *TRGV* could be identified in other recently published novel alleles [19–21]. Mapping of *TRBV* alleles to Luo et al. [19], even allowed us to match the origin of these novel alleles in pmTR resource. This comparison led to the validation of some of our novel alleles from multiple different sources, however, a good mapping of the AS3 alleles to these resources is still not observed. We aim to validate the rest of the alleles using similar approaches to ensure the accuracy of the alleles for studying immune responses and population immunogenetics.

The majority of the novel pmTR alleles were identified in African populations. In total, >90% of these novel alleles are not captured by the IMGT database. Moreover, we found several rare African alleles in all the four *TR* loci. This huge diversity can be explained by the recent genomics-studies in the African populations [25–27]. The sampling of several individuals from many African populations can substantiate our understanding of allelic diversity in *TR* loci. In contrast to *IG* alleles [14], where African-Shared alleles were the second largest group of alleles [14], the Non-African alleles are the second largest group for *TR* alleles. A majority of these Non-African alleles are specific to the Asian populations, suggesting a divergence of *TR* loci, resulting in unique alleles across different ethnicities. We also observed a similar trend in the evolutionary dynamics of the independent *TR* loci represented by the genetic diversity in the PCA plots. Based on the visual inspection of PCA, we observed that the African individuals showed the highest genetic diversity in all the loci. However, we found that the localization of *TR* loci in genome governs the population structures of the four *TR* loci. *TRA* and *TRD* showed similar patterns with the Americans and Europeans being the most diverse, whereas the African populations are the most variable for the *TRB* and *TRG* loci. Here it should be noted that the *TRD* and *TRG* loci are the smallest loci, and, hence, the genetic diversity and the population structure can be affected by the size of the loci.

Gleaning into the gene-specific population distribution of the *TR* alleles, we found a unique pattern in several genes. Some genes had a higher diversity in alleles in preferred populations (e.g., alleles for *TRAV8-4*, TRAV27, *TRBV5-4*, *TRBV7-9* genes comprise African-specific alleles) whereas some tend to be conserved across all the populations (e.g., alleles for *TRAV18, TRAV8-6, TRBV24-1, TRGV1, TRDV1* genes). *D*, *J* and *C* genes in general were conserved suggesting lower level of evolutionary selection pressure on these genes, as compared to the *V* genes, as the alleles for these genes do not harbor population-specific polymorphisms. Moreover, some genes tend to have a huge diversity as compared to other genes (e.g., *TRAV40*, *TRBV7-9*, *TRGV3, TRGV5, TRDV1*). The *TRGV9* and *TRDV2* are heavily expanded in the first few years of life [28]. The high diversity of these genes at population level suggest a specific selection process at the population level as well. All these interesting findings suggest the role of underlying selection mechanisms possibly owing to migratory events of the human populations [29, 30]. The unique diversity of these genes can result in the preferential usage of specific alleles in selective populations ultimately shaping the population-specific immune responses.

Finally, we also ensured that the pmTR alleles from phase 3 dataset were also observed in the NYGC dataset (high coverage and longer reads) that were recently made available [22]. In line with the results mentioned by NYGC [22], we observed higher number of alleles in NYGC dataset as compared to the phase 3 dataset. Although, we could map a majority of the phase 3 alleles in NYGC dataset, the population distribution of the population-specific alleles was heavily affected by the differences in the processing steps between NYGC and phase 3 dataset. This in part could be attributed to the lower accuracy of the allele calls with rare polymorphisms and the accuracy of haplotype phasing per sample in the NYGC variant calling pipeline. As the NYGC reported that the phase 3 study had slightly better haplotype phasing accuracy as compared to the NYGC dataset and we also observed that the population distribution of the phase 3 alleles corroborated better with the previous findings; we used the NYGC dataset as a validation dataset to assess the presence of the phase 3 alleles in long read sequencing data rather than updating the allele calls and population distribution obtained from the NYGC dataset.

Taken together, we meticulously reported curated germline alleles across five ethnicities containing 26 subpopulations, resulting in ~150% more alleles as compared to the known alleles of pmTR database and 73% as compared to the alleles in the IMGT database. Other recent studies [20, 21] have also found a similar higher number of novel alleles. Moreover, one of these studies even reported that 13 novel *TRBV* alleles from pmTR database matched with the novel alleles profiled in their study [20]. Additional benchmarking to similar independent Sanger-sequencing based studies can provide a better measure of reliability to the novel alleles identified in the pmTR database. We believe that the pmTR database adds value to the current resources by enriching population-specific information as well as allelic frequencies to the germline *TR* alleles. This enriched resource can be used for the repertoire studies to understand (population-specific) immune response dynamics.

## Methods

### Data source

The G1K dataset (March, 2019 release; http://www.1000genomes.org; GRCh38 assembly) in the form of phased variant cell format (VCF) was used for retrieving the *TR* germline alleles. Phased variants for GRCh38 (a recent release for the G1K dataset) were used as they were the most recent assembly. However, the GRCh38 genome assembly does not comprise of all the genes profiled in the IMGT database e.g., *TRAJ15*, *TRBV7-8*, *TRBV6-3*, *TRBV4-3*, *TRBV6-9*, *TRBVC*, *TRBV5-8 TRBV3-2* and *TRBD2*. Moreover, there are also some disparities between GRCh37 and GRCh38 version of genome assemblies in terms of *TR* loci. For example, *TRAJ15* and *TRBD2* genes are available in GRCh37 version of the genome. In the release used in this study, G1K project consortium used whole genome (read length >70 bp) and whole exome (read length >68 bp) and microarray genotyping data for the samples to the GRCh38 genome [31]. The full release of the G1K dataset was collected from 2548 cell samples (the trios were not included in the study) from diverse ethnic groups that have a uniform distribution of individuals across populations. The samples are classified in five super populations i.e., Africa, America, East Asia, Europe and South Asia, that are further subdivided into 26 populations (7 African, 4 American, 5 East Asian, 5 European and 5 South Asian populations) with a minimum of 61 and a maximum of 113 samples per population (Table S1). The phased VCF format of the data comprises information of both paternal and maternal chromosomes for each sample.

### Terminology and nomenclature

With the term *“haplotype*” we refer to a gene present on one strand (inherited from a single parent) in one individual. Therefore, there are two haplotypes, one on the paternal and one on the maternal strand, with exactly the same or different polymorphisms. *“Allele”* refers to a haplotype from multiple individuals consisting of the same variants across the complete gene sequence. *“Mutations”* are genetic mutations that occurred to form different alleles (also denoted as allelic variants) of the same gene. The IMGT nomenclature is used to name genes, and this name is extended with a numbering to refer to the different alleles, for example the 01 and 02 alleles of the *TRAV1* gene are referred to as *TRAV1_01*, *TRAV1_02*. IMGT alleles are denoted with an asterix, such as TRAV1*01, TRAV1*02. The alleles were sorted in descending order such that the first allele is supported by the maximum number of haplotypes.

### An automated pipeline to identify germline alleles from G1K data

The pmAlleleFinder pipeline is used to infer the alleles of the genes of interest i.e., *V*, *D*, *J* and *C* genes and RSS from the input VCF file. The pipeline results in a list of alleles for each gene separately along with the population information of each allele in a separate file (Fig. S1). It finds all haplotypes for each gene (5096 haplotypes from 2548 samples), merges them into alleles and counts the haplotype frequency of each such allele. The alleles can be filtered by the user through defining a minimum number of haplotype support. The pipeline is developed in python and R with an additional possibility to automatically identify if pmTR alleles are present in the IMGT database. The pipeline is not limited to the identification of alleles for *TR* genes only and given a phased VCF file the pieline can be used to find population-based alleles for any gene.

### Categorizing pmTR alleles into three groups

Similar to pmIG resource [32], the alleles obtained for *TR* locus from the G1K resource were classified into three major categories (allele set (AS) 1–3):

AS1 (known): G1K alleles with a minimum support of four haplotypes and identified in the IMGT databases. This AS1 allele set has the highest level of confidence as the alleles are observed in the G1K resource as well as in the IMGT database.

AS2 (frequent novel alleles): G1K alleles with a minimum support of 19 haplotype (at least ten individuals). These alleles represent a set of newly identified alleles that are frequent. Similar to pmIG resource, we used 19 haplotypes as cut-off for frequent novel alleles.

AS3 (rare novel alleles): G1K alleles that have a haplotype support between 4 and 18 (supported by two to nine individuals). Despite the rarity of these alleles, we believe that the chance that 4 *identical* haplotypes within 5096 independent haplotypes are caused by sequencing errors is highly unlikely. Unlike pmIG resource, we used a lower threshold here as the *TR* locus is devoid of polymorphisms related to the somatic hypermutations processes that impact *IG* locus owing to the EBV arrested cell lines.

As few of the paralogous *V* genes [24, 33] are highly similar, the mapping of short reads to such genes can be erroneous, influencing the subsequently derived alleles. Called mutations on the alleles of such genes can thus easily be false positives, even after using stringent parameters. Therefore, we denote these genes as OI genes. As these genes can be recognized based on their sequence similarity [23], we generated a neighbor-joining tree for all *V* genes on the *TRA*, *TRB, TRG* and *TRD* loci, separately. The genes sharing a clade with a short branch length, i.e., 0.02, are called OI genes (Fig. S2); and the corresponding alleles OI alleles.

### Filtering out false-positive alleles

The G1K alleles were scrutinized manually: (1) alleles with stop codons were removed from the final set, and (2) alleles within OI genes were removed when they had a mutation that is shared with alleles for the other OI genes as it points toward a mis-alignment of a read (when the mutation is present in the IMGT databases across multiple alleles it is not filtered).

### Validation of the pmTR alleles using NYGC long read data and published literature

Recently, the NYGC released a long read and high coverage data for the G1K 2504 samples and 3202 samples (2504 + 698 trios samples) [22]. Similar to phase 3 dataset, we called alleles for *TR* loci form both NYGC-2504 and NYGC-3202 datasets. Furthermore, we compared the alleles between the phase 3 and both NYGC datasets quantitatively and at the sequence level.

Additionally, several *TR* alleles have been profiled previously in the literature by Luo et al. [19], Lin et al. [21] and Omer et al. [20]. We compared the pmTR alleles with the alleles mentioned in the three literature studies and mentioned the overlap in the supplementary dataset.

### Population annotation of alleles

G1K alleles are annotated with super-population information (Tables S2–S5) into four categories: (1) ALL, present in all super-populations; (2) AFR, only present in Africans; (3) AFR-SHARED, present in African and at least one of the other super-populations, but not all; and (4) NON-AFR, present in at least one of the super-populations but not in Africans.

### Phylogenetic trees for alleles

Maximum Likelihood (ML) trees were built for the alleles using RAxML [34]. The PROTGAMMAJTT model was used to build the trees with 100 bootstraps. The trees were visualized using the iTOL server [35]. The trees taxa were colored as per AS classification; the population level annotation is displayed in binary format and the frequency of alleles as text. A few alleles derived from loci not meant for evaluation were used as an outgroup in all the ML trees.

### pmTR online database

The online pmTR database front-end is made with ReactJS (https://reactjs.org/) in combination with the Neo4j graph database (https://neo4j.com/) back-end to load and display all genes, respective alleles, population frequencies and allele categories (AS1–AS3). Genes can be searched on the basis of their name or nucleotide sequence enabled by a BLAST search [36] (Fig. S3). The pmTR is hosted on https://pmtrig.lumc.nl/.

### Genetic diversity in among four *TR* loci

The VCF file of the complete individual locus, i.e., *TRA* (Chr14 [21621904, 22552132), *TRB* (Chr7 [142299011,142813287]), *TRG* (Chr7 [38240024,38368055]) and *TRD* (Chr14 [22422546,22466577]) was obtained. The complete set of SNPs from the coding and non-coding region from each locus were independently subjected to a PCA using the R Bioconductor package “SNPRelate” [37]. The pairwise population differentiation, quantified by the fixation index (F_ST_), was calculated based on levels of differentiation in polymorphism frequencies across populations. F_ST_ is proportional to the evolutionary branch length between each pair of populations. A Neighbor joining tree was used to visualize the F_ST_ distances between populations.

## Supplementary information

Supplemenatry material

Supplementary dataset

## Data Availability

The source code of the automated tool to identify population-matched alleles and automated mapping to known resources is available via GitHub (https://github.com/JulianDekker/PMalleleFinder). The database is hosted via a website at www.pmTRIG.com.
