# Supplementary material for: pmTR database: population matched (pm) germline allelic variants of T-cell receptor (TR) loci
Source: Genes Immun. 2022 Apr 18;23(2):99–110. doi: 10.1038/s41435-022-00171-x (PMC9042698; doi:10.1038/s41435-022-00171-x)
Supplement: Supplementary file 1 — Supplemenatry material [file 41435_2022_171_MOESM1_ESM.pdf]

pmTR database: population matched (pm) germline allelic variants of T-cell receptor (*TR*) loci

Supplementary Information

Julian Dekker[123], Jacques J.M. van Dongen[1*], Marcel J.T. Reinders[24] and Indu Khatri[12]

Affiliations:

[1]Department of Immunology, Leiden University Medical Center, 2333 ZA Leiden, The Netherlands.

[2] Leiden Computational Biology Center, Leiden University Medical Center, 2333 ZC Leiden, The Netherlands.

[3]Hogeschool, Leiden.

[4] Delft Bioinformatics Lab, Delft University of Technology, 2628 CD Delft, The Netherlands.

*Corresponding author:

Prof. Jacques J.M. van Dongen, MD, PhD

Department of Immunology
Leiden University Medical Center
2333 ZA Leiden
The Netherlands
Email: J.J.M.van_Dongen@lumc.nl

Running Title: pmTR germline allelic database

Keywords: population, germline, allelic variants, *TR* loci, diversity

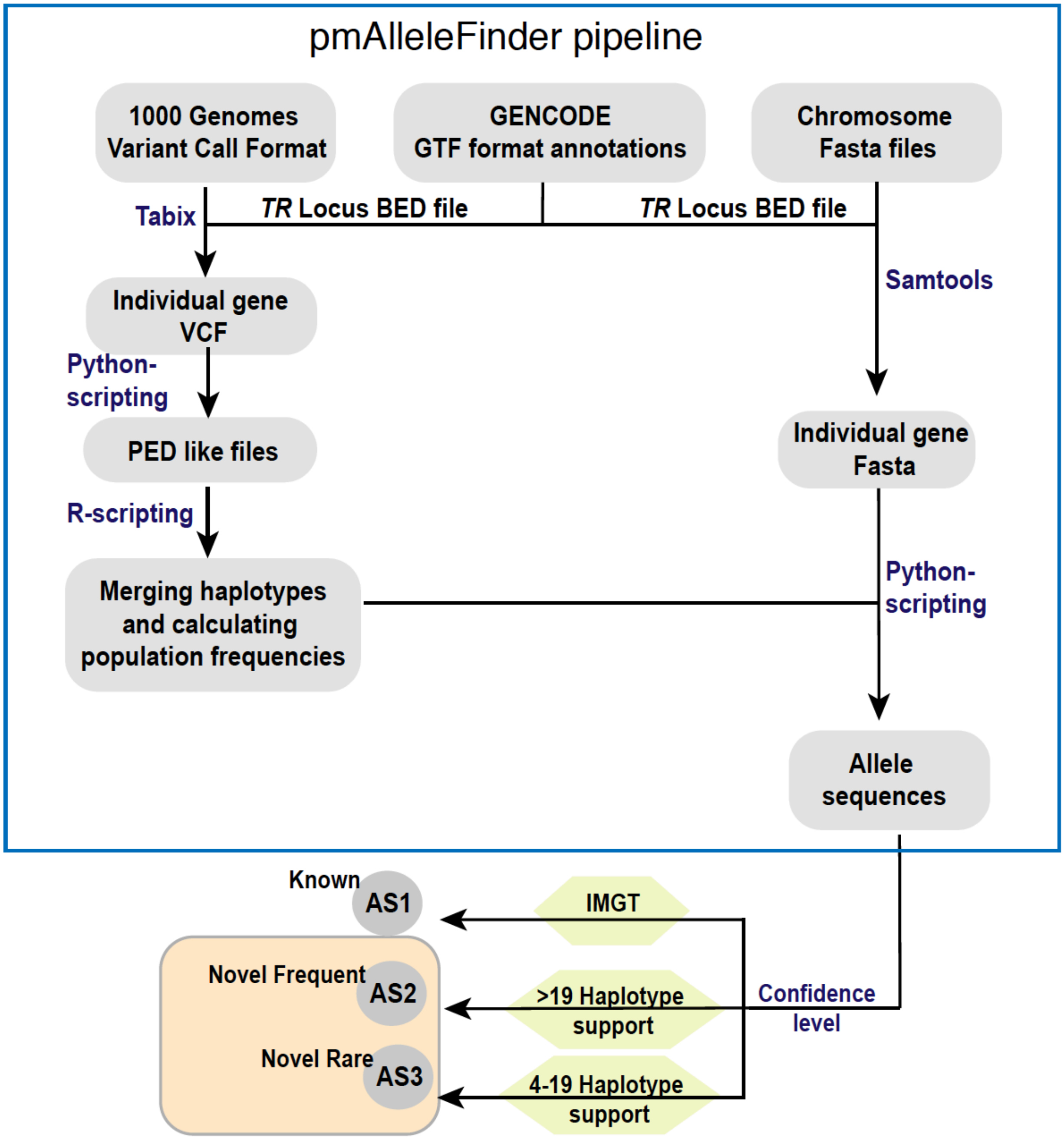

Figure S1: pmAlleleFinder: an automated pipeline to identify population matched alleles. The workflow depicts the VCF, GENCODE GTF format and Chromosome Fasta files are needed as input. The combination of python and R scripts are to identify haplotypes, population frequency and the allele sequences. These sequences are further divided into three categories using automated pipeline developed in Python.

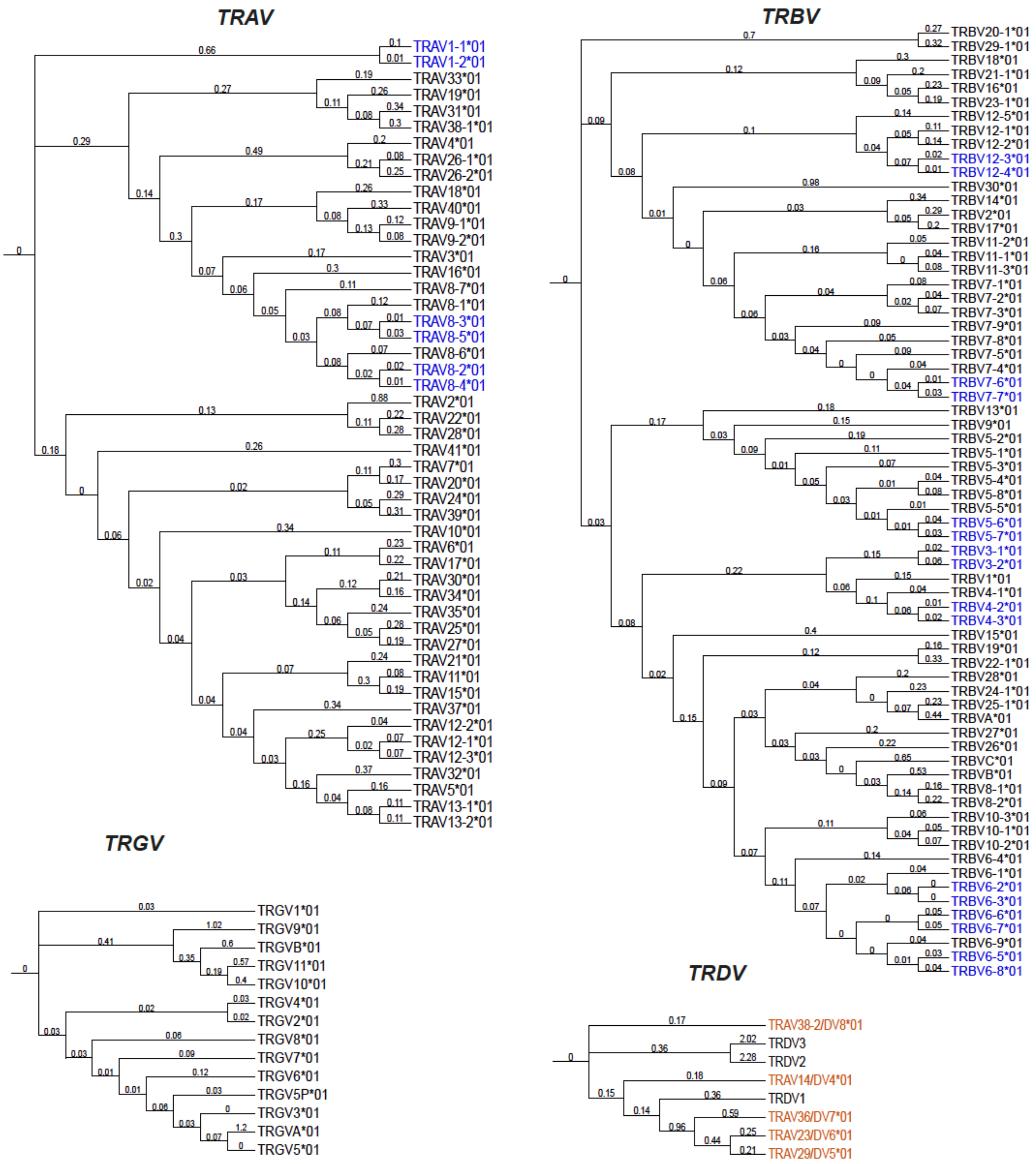

Figure S2: Neighbor Joining tree for the *TRAV*, *TRBV*, *TRGV* and *TRDV* genes from IMGT database to identify duplicated genes. The genes separated by a small distance are considered as operationally indistinguishing genes that are marked in blue in the tree. The alleles from these genes are scrutinized manually for shared mutating positions. Please note that the nomenclature of *TRDV4-8* genes correspond to *TRAV* genes as: *TRDV4* is *TRAV14*, *TRDV5* is *TRAV29*, *TRDV6* is *TRAV23*, *TRDV7* is *TRAV36* and *TRDV8* is *TRAV38-2* (colored in orange).

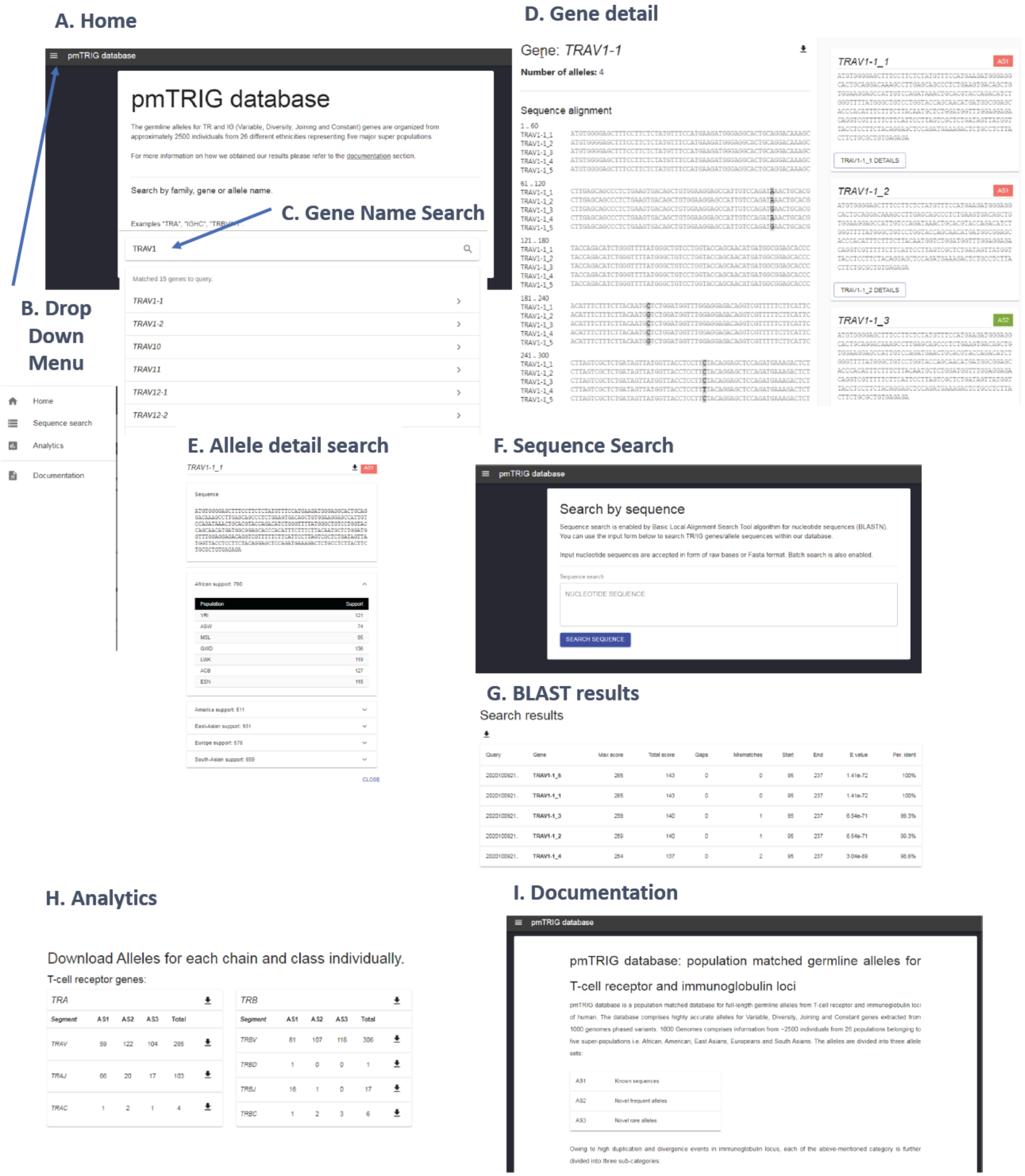

Figure S3: pmTRIG database: The database with organized population matched germline alleles, identified from G1K samples, for the *TR* and *IG* loci. All the different components of the website are shown i.e. **A)** Home screen; **B)** Drop-down Menu; **C)** Box with gene name search; **D)** result for a gene search; **E)** Information provided for each allele; **F)** Sequence search screen; **G)** Output of sequence search in BLAST format; **H)** Analytics page providing downloads of genes for each category for each locus; and **I)** Documentation page for the database.
